# Self-Monitoring of Blood Glucose to Assess Dawn Phenomenon in Chinese People with Type 2 Diabetes Mellitus

**DOI:** 10.1155/2017/7174958

**Published:** 2017-03-21

**Authors:** Wen Wu, Yuxin Huang, Jieyuzhen Qiu, Jiao Sun, Haidong Wang

**Affiliations:** ^1^Department of Endocrinology, Huadong Hospital Affiliated to Fudan University, 221 Yananxi Road, Shanghai 200040, China; ^2^Shanghai Key Laboratory of Clinical Geriatric Medicine, 221 Yananxi Road, Shanghai 200040, China

## Abstract

*Aims*. We investigated whether self-monitoring of blood glucose could be used to assess dawn phenomenon in Chinese people with type 2 diabetes mellitus (T2DM). *Methods*. A total of 306 people with T2DM underwent continuous glucose monitoring and self-monitoring of blood glucose for 72 h. A linear model was used to fit the optimal linear formula of the magnitude of dawn phenomenon (ΔDawn) and self-monitoring of blood glucose values. *Results*. The prevalence of dawn phenomenon was similar within different oral antidiabetic drug groups (42.5%, 31.5%, and 40.9%, *P* = 0.216). Multiple variable linear regression showed that prebreakfast, prelunch, and predinner glucose measurements were independently and significantly correlated with ΔDawn. The linear formula between ΔDawn and blood glucose was as follows: ΔDawn (mg/dL) = 0.557 × prebreakfast − 0.065 × prelunch − 0.164 × predinner − 20.894 (mg/dL) (adjusted *R*^2^ = 0.302, *P* = 0.000). *Conclusions*. Dawn phenomenon could be partly assessed by blood glucose self-monitoring in Chinese people with T2DM using the abovementioned formula. The incidence of dawn phenomenon was similar among patients in different oral antidiabetic drug groups.

## 1. Introduction

“Dawn phenomenon,” first proposed by Schmidt in 1981, describes a spontaneous increase in blood glucose concentration or insulin requirements during the early morning that occurs in the absence of hypoglycaemia [1]. Continuous glucose monitoring system (CGMS), which has been widely used for decades, effectively improves the qualitative and quantitative detection of dawn phenomenon. Using CGMS, dawn phenomenon is determined using the absolute differences between the nocturnal glucose nadir and the prebreakfast glucose values at a threshold of 20 mg/dL [2–4]. Monnier et al. showed that the approximate impact of the dawn phenomenon on HbA_1c_ level was 0.4%, while its impact on averaged 24 h mean glucose concentrations was 12.4 mg/dL [3]. Although dawn phenomenon is not considered a main control target in the management of T2DM, we should not ignore it. It is very likely that the correlation between nocturnal nadir and dawn phenomenon magnitude can be confirmed only by using CGMS; however, CGMS is not widely used in endocrinology departments, and its 3-day usage period is inconvenient for patients. Moreover, CGMS remains costlier than devices used for self-monitoring of blood glucose (SMBG).

In 2015, Monnier et al. [5] published a simple method for assessing and quantifying the presence of the dawn phenomenon using SMBG. They had deduced a formula between magnitude of dawn phenomenon and SMBG as follows: *Y* (magnitude of dawn phenomenon, mg/dL) = 0.49 × (prebreakfast glucose average prelunch and predinner glucose, mg/dL) + 15 (mg/dL) [5]. In addition, receiver operating characteristic curve analysis demonstrated that prebreakfast glucose minus the average prelunch and predinner cutoff value of 10 mg/dL could predict the dawn phenomenon at a threshold of 20 mg/dL [5]. This formula might be a practical, feasible, and reliable method for evaluating dawn phenomenon using SMBG. However, we could not ignore that the study was performed in France and most of the participants were Caucasian. It is well known that Chinese people with T2DM present with lower beta-cell function and higher postprandial blood glucose than Caucasian people with T2DM, although these people matched adequately in age, body mass index, and gender in these studies [6, 7]. Therefore, it is of interest to determine whether this formula is also suitable for Chinese people. It has been reported that the frequency of dawn phenomenon was 33.3% to 78.8% in Chinese people with T2DM [8, 9]. But to our knowledge, sample size in these studies was small. Few high-quality research studies concerning dawn phenomenon in Chinese population have been published. Thus, we felt it imperative that we assess this issue. Here, we investigated whether the self-monitoring of blood glucose could be used to assess dawn phenomenon in Chinese people with T2DM.

## 2. Materials and Methods

### 2.1. Study Subjects

All participants were recruited from the Department of Endocrinology, Huadong Hospital, which is affiliated with Fudan University, Shanghai, People's Republic of China, between May 2014 and March 2016. A total of 320 subjects with T2DM were enrolled in the study according to the protocol. The study protocol was approved by the Huadong Hospital Ethics Committee. All procedures involving human participants were performed in accordance with the ethical standards of the institutional research committee and the 1964 Helsinki declaration. Informed consent was obtained from all individual participants included in the study. All enrolled people were identified by participant numbers in the database to ensure anonymity.

### 2.2. Inclusion Criteria

The inclusion criteria were as follows: (1) diagnosed with T2DM according to the 2014 American Diabetes Association criteria [10]; (2) age > 40 years; and (3) stable treatment with oral hypoglycaemic agents (including metformin, thiazolidinediones, sulfonylureas, and glinides) for at least 3 months. Participants were divided into three groups by treatment modality: group 1, insulin sensitizers (metformin or thiazolidinediones); group 2, insulin secretagogues (sulfonylureas or glinides); and group 3, one insulin secretagogue in combination with one insulin sensitizer.

### 2.3. Exclusion Criteria

The exclusion criteria were as follows: (1) received insulin therapy in 3 months prior to the study; (2) HbA_1c_level > 8.5% (69 mmol/mol); (3) current diabetic ketoacidosis or hyperosmolar coma; (4) current cardiovascular disease or other serious disease; (5) current hypoglycaemia or suspected hypoglycaemia; (6) creatinine clearance rate < 45 mL/min; (7) impaired liver function (liver enzymes more than twice the upper limit of normal); (8) poor medication compliance; and (9) reluctant to undergo CGMS monitoring.

### 2.4. Clinical Investigations and Laboratory Determinations

All patients were instructed to maintain their recommended medication and exercise programs. Following a 10 h overnight fast, anthropometric and blood pressure data and serum and plasma samples were collected. Biochemical measurements of plasma glucose, insulin, HbA_1c_, and serum lipids were performed in a central laboratory. HbA_1c_ levels were determined using a high-performance liquid chromatography assay. Insulin sensitivity was calculated as the homeostatic model assessment of insulin resistance index (HOMA-IR) using the HOMA calculator (Headington, Oxford, UK) (http://www.dtu.ox.ac.uk).

### 2.5. Medical Nutrition Therapy

All participants with T2DM received individualized medical nutrition therapy from registered nutritionists. Energy intake was assumed to be equal to energy expenditure, and carbohydrates provided 50% of the total daily energy intake. Breakfast, lunch, and dinner were calorically divided as 1 : 2 : 2. The three daily meals were required to be ingested between 7:00 and 7:30, 11:00 and 11:30, and 17:00 and 17:30, respectively. Furthermore, the participants were not allowed to consume any snacks between meals.

### 2.6. CGMS and SMBG

All 320 patients with diabetes underwent 72 h CGMS monitoring (MiniMed system, Medtronic Inc., USA). The participants were obliged to input their capillary blood glucose four times a day to calibrate the CGMS. The CGMS sensor was installed on day 0 and removed on day 3. The data provided by the CGMS were obtained during days 1 and 2 to avoid any interference due to sensor installation and removal. Furthermore, the data recorded on days 1 and 2 were averaged to avoid bias. The nocturnal nadir glucose level was quantified by the averaged CGMS data of days 1 and 2. The magnitude of dawn phenomenon (ΔDawn) was quantified by its absolute change from the nocturnal nadir to prebreakfast glucose level, and the dawn phenomenon threshold was set at 20 mg/dL (1.11 mmol/L) according to Monnier et al. [3]. If all overnight glucose measurements were higher than the prebreakfast measurement, ΔDawn was considered as 0 [5].

SMBG was synchronized with CGMS for 72 h. Capillary blood glucose was tested five times a day: between 6:45 and 7:00 (prebreakfast, preBF), 9:00 and 9:15 (postbreakfast, postBF), 10:45 and 11:00 (prelunch, preL), 16:45 and 17:00 (predinner, preD), and 21:15 and 21:30 (bedtime). PreLD was calculated as the average preL and preD value. In addition, ΔpreBF − LD was calculated as the difference between preBF and preLD as mentioned by Monnier et al. The average of the SMBG data from days 1 and 2 was also calculated to avoid bias. It should be noted that the ΔDawn level was determined only by CGMS because the nocturnal glucose nadir could not be obtained by SMBG. Other glucose levels were determined by SMBG.

### 2.7. Statistical Analysis

Continuous variables are expressed as mean ± SD and were determined using analysis of variance testing for normally distributed data. Categorical variables are expressed as numbers (%) and were analysed using the chi-square test and Fisher's exact test. Relationships between SMBG values (preBF, postBF, preL, preD, bedtime, preLD, and ΔpreBF − LD) and nocturnal nadir glucose were calculated using Pearson's coefficient. All *P* values were two-tailed, and those < 0.05 were considered significant. A backward linear model was used to examine the independent relationship between ΔDawn and SMBG values and fit the optimal linear formula between the ΔDawn and SMBG values. Statistical analyses were performed with SPSS version 13.0 software (SPSS Inc., Chicago, IL, USA). All figures were created using GraphPad Prism 5 (GraphPad Software Inc., San Diego, CA, USA).

## 3. Results and Discussion

### 3.1. Clinical Characteristics

A total of 320 people with T2DM were enrolled in the study. Fourteen subjects who presented with hypoglycaemia (blood glucose < 70 mg/dL) or suspected hypoglycaemia during the CGMS monitoring were excluded from the study to avoid the Somogyi effect. The other 306 participants completed the entire protocol. The clinical characteristics of these participants are summarized in Table 1. The included participants were separated into three groups by treatment modality: group 1, treated with insulin sensitizers (*n* = 80); group 2, treated with insulin secretagogues (*n* = 111); and group 3, treated with insulin secretagogues in combination with insulin sensitizers (*n* = 115). The mean age was 65.8 ± 11.1 years in group 1, 64.1 ± 12.0 years in group 2, and 68.42 ± 9.2 years in group 3 (*P* = 0.011). The mean duration of diabetes was 8.5 ± 4.1 years in group 1, 10.0 ± 7.5 years in group 2, and 13.4 ± 6.6 years in group 3. Individuals in group 3 had a longer mean disease duration than patients in the other two groups (*P* = 0.000). Sex, body mass index, mean daily energy intake, mean daily carbohydrate intake, and lipid profile did not differ significantly among the three groups. Individuals in group 3 had a higher mean HbA_1c_ level (*P* = 0.000) and lower mean fasting insulin and HOMA-IR levels (*P* = 0.002 and *P* = 0.033, resp.) than those in the other two groups.

### 3.2. Analysis of SMBG and Dawn Phenomenon

**Figure 1:**
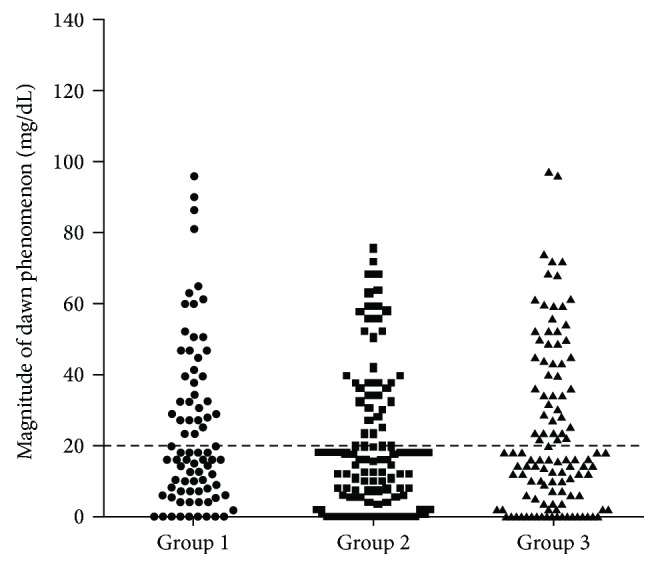
Magnitude of the dawn phenomenon (ΔDawn) in three groups. Group 1, treated with insulin sensitizers (black circle); group 2, treated with insulin secretagogues (black square); and group 3, treated with insulin secretagogues in combination with insulin sensitizers (black triangle). When the threshold of ΔDawn was set at 20 mg/dL, the prevalence of dawn phenomenon in groups 1–3 was 42.5%, 31.5%, and 40.9%, respectively (*P* = 0.216).

All patients in this study underwent 3-day CGMS and SMBG. The SMBG and dawn phenomenon magnitude (ΔDawn) data are summarized in Table 1. The mean glucose levels at preBF, postBF, preL, preD, and bedtime were 136.3 ± 26.3 mg/dL, 166.6 ± 41.5 mg/dL, 133.7 ± 37.8 mg/dL, 146.1 ± 39.7 mg/dL, and 151.7 ± 38.9 mg/dL, respectively. People in group 3 showed a higher mean preD glucose level than patients in the other two groups (*P* = 0.000). The mean glucose level at nocturnal nadir was 114.5 ± 25.8 mg/dL. The ΔDawn value calculated as preBF minus the nocturnal nadir was 22.4 ± 20.8 mg/dL in the total population and 24.7 ± 22.9, 20.2 ± 19.8, and 22.9 ± 22.1 mg/dL in the three groups (*P* = 0.364). When the ΔDawn threshold was set at 20 mg/dL, 116 of 306 (37.9%) people with T2DM suffered from dawn phenomenon. The prevalence of dawn phenomenon in groups 1–3 was 42.5%, 31.5%, and 40.9%, respectively. There was no statistical difference in the prevalence of dawn phenomenon among the three groups categorized by antidiabetic treatment type (*P* = 0.216; Figure 1).

### 3.3. Correlation Analysis

**Figure 2:**
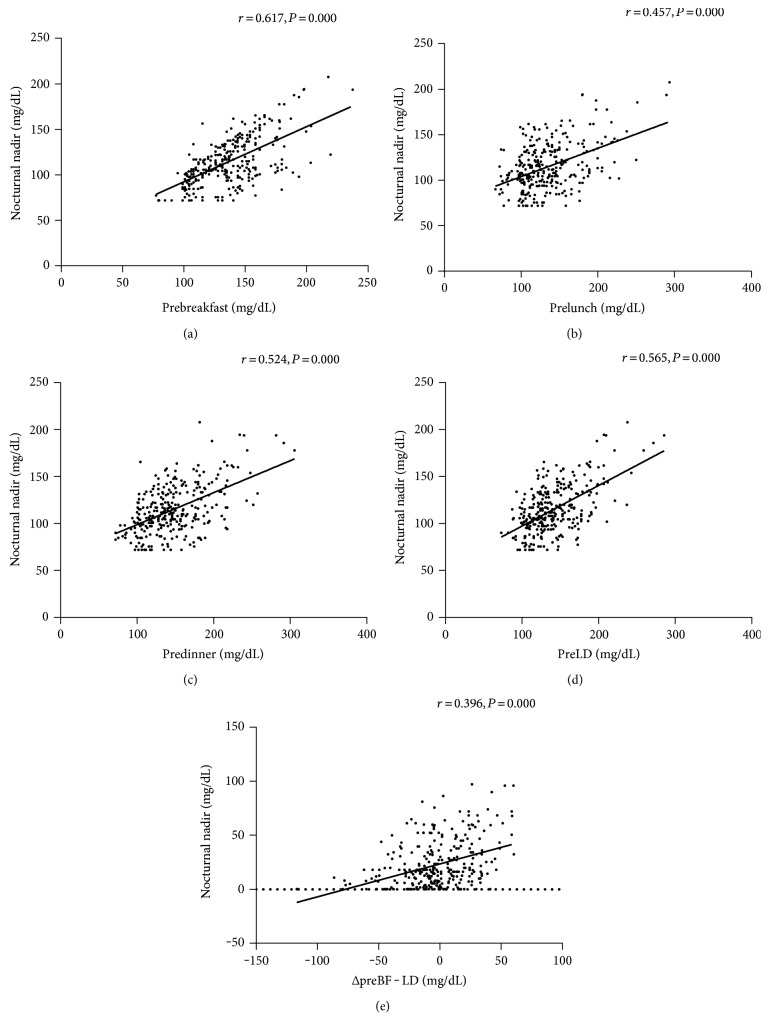
Relationships between nocturnal nadir and glucose values. (a) Relationship between nocturnal nadir and prebreakfast glucose. (b) Relationship between nocturnal nadir and prelunch glucose. (c) Relationship between nocturnal nadir and predinner glucose. (d) Relationship between nocturnal nadir and average of preL and preD glucose (preLD). (e) Relationship between the magnitude of dawn phenomenon (ΔDawn) and the calculated difference between prebreakfast and preLD (ΔpreBF − LD). The relationship is ΔDawn  (mg/dL) = 0.306 × ΔpreBF − LD + 23.52  (mg/dL) (*r* = 0.396, *P* = 0.000).

Correlation analysis showed that the nocturnal glucose nadir was significantly correlated with the following SMBG levels: preBF, *r* = 0.617, *P* = 0.000; postBF, *r* = 0.406, *P* = 0.000; preL, *r* = 0.457, *P* = 0.000; preD, *r* = 0.524, *P* = 0.000; bedtime, *r* = 0.407, *P* = 0.000; and preLD (average of preL and preD), *r* = 0.565, *P* = 0.000. Accordingly, the preBF, preL, preD, and preLD glucose levels were more closely correlated with nocturnal nadir (Figure 2). However, the correlation weakened when ΔDawn was correlated with the calculated difference between preBF and preLD (ΔpreBF − LD): *r* = 0.396, *P* = 0.000 (Figure 2).

### 3.4. Linear Regression Model

Since the preBF, preL, preD, and preLD glucose levels were more closely correlated with nocturnal nadir than the other levels, our full linear regression model contained these four points of glucose as independent variables. Variables were entered into the model if the significance of the *F* value was <0.05 and removed if it was >0.10. Multiple variable linear regression showed that preBF, preL, and preD were independently and significantly correlated with ΔDawn level: preBF, *β* = 0.557, *P* = 0.000; preL, *β* = −0.065, *P* = 0.038; and preD, *β* = −0.164, *P* = 0.000. The linear formula between ΔDawn and SMBG was ΔDawn (mg/dL) = 0.557 × preBF − 0.065 × preL − 0.164 × preD − 20.894 (mg/dL) (adjusted *R*^2^ = 0.302, *P* = 0.000). As the formula calculated by Monnier et al. was ΔDawn (mg/dL) = 0.49 × ΔpreBF − LD + 15 (mg/dL), we also created a similar linear regression between ΔDawn and ΔpreBF − LD. The linear formula between ΔDawn and  ΔpreBF  − LD was ΔDawn (mg/dL) = 0.306 × ΔpreBF − LD + 23.52 (mg/dL) (adjusted *R*^2^ = 0.154, *P* = 0.000).

### 3.5. Discussion

In recent decades, studies have revealed that dawn phenomenon could only be confirmed by CGMS because the nocturnal nadir could not be easily observed. However, Monnier et al. [5] published a method of assessing dawn phenomenon using SMBG instead of CGMS. Self-monitoring of preprandial glucose values at each of the three meals could predict the occurrence of dawn phenomenon. The formula was as follows: *Y* (ΔDawn, mg/dL) = 0.49 × (ΔpreBF − LD, mg/dL) + 15 (mg/dL). This method might be able to replace CGMS to confirm dawn phenomenon. It was a feasible and cost-efficient and could easily be used in basic-level hospitals. In our study, focusing on 306 Chinese people with T2DM, we created a similar linear regression between ΔDawn and ΔpreBF − LD using the following linear formula: ΔDawn (mg/dL) = 0.306 × ΔpreBF − LD + 23.52 (mg/dL). Unfortunately the adjusted *R*^2^ was only 0.154 in this linear regression. Thus, we created another linear regression between SMBG and ΔDawn level: ΔDawn (mg/dL) = 0.557 × preBF − 0.065 × preL − 0.164 × preD − 20.894 (mg/dL). Although it was a bit more complicated than the formula of Monnier et al., the adjusted *R*^2^ increased to 0.302.

There may be a few reasons for these differences. First, the ancestors of Caucasian were nomadic people, while the ancestors of Chinese people were agricultural people [11]. Throughout history, carbohydrates have been the mainstay of the modern Asian diet [12]. According to the Food and Agriculture Organization [13], the average supply of protein of animal origin between 2009 and 2011 was 72 g in France versus only 37 g in China. However, the share of dietary energy supply derived from cereals, roots, and tubers was 29% in France and 52% in China. Ethnic dietary differences might be another influential factor. Second, the United Kingdom Prospective Diabetes Study reported that by the time a patient receives the diagnosis of T2DM, pancreatic beta-cell function has already been reduced by half [14]. Beta-cell dysfunction plays a critical role in the progression of hyperglycaemia in T2DM. Several studies have suggested that beta-cell function is fairly lower in Asians than that in Caucasians [15–18] and Asians have less beta-cell regenerative capacity than Caucasians [19]. Beta-cell dysfunction is concerned with postprandial blood glucose increase and glucose excursion [19]. Thus, in Asian patients with diabetes, the extreme increase in postprandial blood glucose is a particularly significant characteristic. Several studies have reported that Asians demonstrated higher postprandial glucose levels [20, 21], higher HbA_1c_ levels [22], and increased risk of diabetes-related complications than Caucasians [23, 24]. Even when provided the same food, Asian people presented significantly higher glycaemic responses to meals than Caucasians [25]. Moreover, Chinese women usually presents with higher dietary glycaemic loads and glycaemic indexes than Caucasian women [26].

In Monnier et al.'s [5] research, the mean glucose levels were 131.4 ± 2.5 mg/dL for preBF, 126.5 ± 2.7 mg/dL for preL, and 121.1 ± 2.9 mg/dL for preD. The averaged preL and preD glucose levels (preLD) were strongly correlated with nocturnal nadir (*r* = 0.83, *P* < 0.0001). As such, they used preBF minus preLD (Δ − LD) to predict the ΔDawn (preBF − nocturnal nadir) using the formula *Y* (ΔDawn, mg/dL) = 0.49 × (ΔpreBF − LD, mg/dL) + 15 (mg/dL). Here, we provided the same proportion of carbohydrates of total daily energy intake as that reported by Monnier et al. [5]. However, the mean preBF, preL, and preD glucose levels were 136.3 ± 26.3 mg/dL, 133.7 ± 37.8 mg/dL, and 146.1 ± 39.7 mg/dL, respectively. The daytime glucose levels reported here are higher than those reported by Monnier, and mean premeal glucose levels presented a chaos profile. Nocturnal nadir in our study was more weakly correlated with preLD glucose level (*r* = 0.565, *P* = 0.000). Thus, the adjusted *R*^2^ was only 0.154 in the linear regression between ΔDawn and ΔpreBF − LD. The difference might be caused by low beta-cell function and high postprandial glucose levels in the Chinese population.

The dawn phenomenon mechanism remains unclear. It is generally believed that dawn phenomenon is a result of pancreas islet beta-cell dysfunction, which involves increased endogenous glucose production, persistent insulin resistance, and hepatic glucose output in people with type 1 diabetes mellitus or T2DM [27, 28]. A study in Chinese people with type 2 diabetes proved that dawn phenomenon is closely associated with obesity and insulin resistance. The frequency of dawn phenomenon increases with body mass index [8]. Another cross-sectional study in China showed that sleep disorders were associated with dawn phenomenon [9]. In addition, elevated level of growth hormone in the nocturnal hours and increased levels of cortisol in the early morning lead to insulin signal conduction system damage and enhanced fat decomposition as well as further increased insulin resistance throughout the whole body. Liver glycogen decomposition, increased endogenous gluconeogenesis, and decreased peripheral tissue insulin action could eventually cause the dawn phenomenon [29].

The drawbacks of dawn phenomenon have been well published. The approximate impact of dawn phenomenon on HbA_1c_ level was 0.4%, while that on mean 24 h glucose concentration was 12.4 mg/dL [3]. Furthermore, Monnier et al. studied dawn phenomenon in three groups of people with T2DM (on diet only, on insulin sensitizers alone, on insulin secretagogues alone, or on insulin secretagogues in combination with insulin sensitizers) using CGMS and reported similar ΔDawn levels among the three groups [5]. We observed similar results. The prevalence of dawn phenomenon in the three groups (treated with insulin sensitizers, treated with insulin secretagogues, and treated with insulin secretagogues plus insulin sensitizers) was 42.5%, 31.5%, and 40.9%, respectively. These results are very interesting. Although mean HbA_1c_ differed among the three groups, there was no statistically significant difference in the prevalence of dawn phenomenon among them. Oral antidiabetic medications might not be very effective at controlling dawn phenomenon, even when used as combined therapy. Further studies are needed to verify this hypothesis.

## 4. Conclusion

In conclusion, the current study revealed that dawn phenomenon could be assessed by SMBG in Chinese people with T2DM using the following formula: ΔDawn (mg/dL) = 0.557 × preBF − 0.065 × preL − 0.164 × preD − 20.894 (mg/dL). This might be a feasible and reliable method of evaluating dawn phenomenon using SMBG. Furthermore, the incidence of dawn phenomenon was similar among different oral antidiabetic drug groups, even when given as combined therapies.

## Figures and Tables

**Table 1:** Characteristics and glucose levels of total population and groups treated with insulin sensitizers, insulin secretagogues, and insulin sensitizers plus insulin secretagogues.

Variables	Total	Insulin sensitizers	Insulin secretagogues	Sensitizers + secretagogues	^a^ *P* value
Number (M/F)	306 (191/115)	80 (56/24)	111 (68/43)	115 (67/48)	0.238
Age (years)	66.2 ± 10.9	65.8 ± 11.1	64.1 ± 12.0	68.42 ± 9.2	0.011
BMI (kg/m^2^)	25.3 ± 2.2	26.7 ± 2.5	24.6 ± 2.6	24.4 ± 2.1	0.054
Duration (years)	10.9 ± 6.7	8.5 ± 4.1	10.0 ± 7.5	13.4 ± 6.6	0.000
Mean daily energy intake (kcal)	1498.5 ± 202.1	1528.2 ± 198.6	1505.2 ± 196.2	1472.0 ± 208.4	0.151
Mean daily carbohydrate intake (g)	187.3 ± 25.3	191.0 ± 24.8	188.1 ± 24.5	184.0 ± 26.1	0.151
Fasting insulin (*μ*U/mL)	9.9 ± 6.2	12.8 ± 7.4	9.5 ± 6.4	8.2 ± 5.6	0.002
HOMA-IR	3.3 ± 3.1	4.0 ± 3.9	3.1 ± 2.2	2.8 ± 2.2	0.033
HbA_1c_ (%)	7.1 ± 0.9	6.7 ± 0.9	7.1 ± 0.9	7.3 ± 0.8	0.000
Triglyceride (mmol/L)	1.6 ± 1.0	1.5 ± 0.7	1.6 ± 1.2	1.5 ± 1.0	0.617
Total cholesterol (mmol/L)	4.3 ± 1.1	4.4 ± 1.2	4.4 ± 1.1	4.2 ± 1.0	0.209
PreBF (mg/dL)	136.3 ± 26.3	132.1 ± 26.7	136.6 ± 24.0	138.9 ± 28.1	0.197
PostBF (mg/dL)	166.6 ± 41.5	159.0 ± 46.2	166.0 ± 38.7	172.5 ± 40.0	0.082
PreL (mg/dL)	133.7 ± 37.8	128.8 ± 38.9	133.9 ± 35.0	137.1 ± 39.4	0.322
PreD (mg/dL)	146.1 ± 39.7	132.6 ± 38.5	144.9 ± 36.0	157.1 ± 41.3	0.000
Bedtime (mg/dL)	151.7 ± 38.9	143.7 ± 40.1	151.7 ± 37.8	157.4 ± 38.6	0.056
Nadir (mg/dL)	114.5 ± 25.8	107.7 ± 22.7	116.6 ± 24.6	117.2 ± 28.1	0.022
ΔDawn (mg/dL)	22.4 ± 20.8	24.7 ± 22.9	20.2 ± 19.8	22.9 ± 22.1	0.364
Dawn phenomenon (%)	116 (37.9)	34 (42.5)	35 (31.5)	47 (40.9)	0.216

Data are means ± SD or number (percentage).

^a^ *P* value among groups.

BMI: body mass index; HOMA-IR: homeostatic model assessment of insulin resistance; preBF: prebreakfast; postBF: postbreakfast; preL: prelunch; preD: predinner; ΔDawn, difference between prebreakfast and nocturnal nadir glucose values; dawn phenomenon, ΔDawn > 20 mg/dL.
